# The Potential of Influenza HA-Specific Immunity in Mitigating Lethality of Postinfluenza Pneumococcal Infections

**DOI:** 10.3390/vaccines7040187

**Published:** 2019-11-17

**Authors:** Miriam Klausberger, Irina A. Leneva, Irina N. Falynskova, Kirill Vasiliev, Alexander V. Poddubikov, Claudia Lindner, Nadezhda P. Kartaschova, Oxana A. Svitich, Marina Stukova, Reingard Grabherr, Andrej Egorov

**Affiliations:** 1 Department of Biotechnology, University of Natural Resources and Life Sciences (BOKU), 1190 Vienna, Austria; claudia.lindner@evercyte.com; 2 Department of Virology, I. Mechnikov Research Institute for Vaccines and Sera, Moscow 105064, Russia; wnyfd385@yandex.ru (I.A.L.); falynskova@mail.ru (I.N.F.); nadezdakartasova10571@gmail.com (N.P.K.); svitichoa@yandex.ru (O.A.S.); aevirol@gmail.com (A.E.); 3 Smorodintsev Research Institute of Influenza, St. Petersburg 197376, Russia; kirillv5@yandex.ru (K.V.); stukova@influenza.spb.ru (M.S.); 4 Department of Microbiology, I. Mechnikov Research Institute for Vaccines and Sera, Moscow 105064, Russia; poddubikov@yandex.ru

**Keywords:** Influenza, *Streptococcus pneumoniae*, secondary bacterial infection, off-target vaccine effects, anti-HA immunity, type I/II interferon, VLP vaccine, baculovirus inactivation

## Abstract

Influenza virus infections pre-dispose an individual to secondary pneumococcal infections, which represent a serious public health concern. Matching influenza vaccination was demonstrated helpful in preventing postinfluenza bacterial infections and associated illnesses in humans. Yet, the impact of influenza hemagglutinin (HA)-specific immunity alone in this dual-infection scenario remains elusive. In the present study, we assessed the protective effect of neutralizing and non-neutralizing anti-hemagglutinin immunity in a BALB/c influenza-pneumococcus superinfection model. Our immunogens were insect cell-expressed hemagglutinin-Gag virus-like particles that had been differentially-treated for the inactivation of bioprocess-related baculovirus impurities. We evaluated the potential of several formulations to restrain the primary infection with vaccine-matched or -mismatched influenza strains and secondary bacterial replication. In addition, we investigated the effect of anti-HA immunity on the interferon status in mouse lungs prior to bacterial challenge. In our experimental setup, neutralizing anti-HA immunity provided significant but incomplete protection from postinfluenza bacterial superinfection, despite effective control of viral replication. In view of this, it was surprising to observe a survival advantage with non-neutralizing adaptive immunity when using a heterologous viral challenge strain. Our findings suggest that both neutralizing and non-neutralizing anti-HA immunity can reduce disease and mortality caused by postinfluenza pneumococcal infections.

## 1. Introduction

A leading cause of the mortality observed during both seasonal and pandemic influenza outbreaks can be attributed to secondary respiratory infections with bacteria, such as *Streptococcus pneumoniae* (S. pneumoniae, pneumococcus) [1]. Pneumococcus is a frequent commensal of the human upper respiratory tract of healthy individuals, with the highest prevalence (up to 50%) in children younger than two years of age [2]. Influenza and pneumococcal infections follow a winter seasonality pattern [3]. This characteristic increases the likelihood for combined or sequential infections, which manifest as more severe illnesses with higher mortality rates than disease caused by either pathogen alone [4]. Murine studies demonstrated that an influenza virus infection increases the susceptibility to subsequent pneumococcal infection and revealed potential mechanisms involved. There is strong evidence that virus-mediated activation of innate immunity plays a decisive role in rendering an influenza-infected individual less capable of mounting a proper immune response towards a secondary bacterial invader [5,6,7,8,9]. In this regard, expression of the innate cytokines type I (α/β) and type II (γ) interferon (IFN) in response to viral infection can attenuate the phagocytic function of tissue-resident alveolar macrophages (AMs) [10,11] or impair the recruitment of neutrophils [12] and natural killer (NK) cells [13] to the site of infection. Moreover, type I IFNs were associated with the negative regulation of unconventional T cells (γδ T cells) by blocking the expression of cytokines (i.e., interleukin-17A, IL-17A) that are pivotal in initiating effective antibacterial innate immune responses [5,14].

One way to prevent postinfluenza pneumococcal complications would be by means of prophylactical measures against the bacterial pathogen. There are, however, indications that pneumococcal-specific vaccine-induced immunity is not effective in the context of viral-bacterial infections [15,16]. In addition, marketed pneumococcal vaccines provide serotype-specific immunity and cover only a fraction (max. 23) out of 98 currently known serotypes [17]. With the widespread introduction of childhood pneumococcal immunization programs vaccine serotypes in circulation have been rapidly replaced by non-vaccine serotypes, which compromises the benefit of implemented programs [18]. There is a limited but growing number of studies available that acknowledge the protective role of influenza vaccination in the context of secondary bacterial infections (SBIs) in the mouse model and in humans [19,20,21,22]. Influenza vaccination predominantly focuses on the induction of antibodies towards the head domain of the influenza hemagglutinin (HA). Such antibodies effectively prevent an infection, but the rapid antigenic drift of the protein may render elicited immunity ineffective [23]. Still, mismatched influenza vaccines prime non-neutralizing immunity that do not prevent an infection but can reduce illness and mortality [24,25,26].

In the present study, we investigated the distinct role of neutralizing and non-neutralizing anti-HA immunity in the protection from postinfluenza pneumococcal disease and mortality in a murine BALB/c superinfection model. We employed different vaccine preparations based on Gag-virus-like particles (Gag-VLPs) containing the influenza HA of A/PR/8/34 (H1N1) that were expressed in insect cells using the baculovirus expression vector system. To abolish potential immune-modulating effects of residual baculovirus (BV) in the preparations, we employed two alternative chemicals (β-propiolactone or binary ethylenimine) for viral inactivation. Vaccine efficacy was evaluated after infection with antigenically distinct H1N1 viruses followed by a secondary pneumococcal challenge. We tested the effect of immunization on the host IFN response after viral infection and on disease exacerbation after secondary bacterial infection.

## 2. Materials and Methods 

### 2.1. Ethics Statement

All animal experiments were conducted in strict accordance with the “Rules for laboratory practice in the Russian Federation” of the Ministry of Health of Russia (23.08.2010 No. 708h) and were approved by the Institutional Animal Care and Use Committee (IACUC) of the I. Mechnikov Research Institute for Vaccines and Sera, Moscow Russia (28/01/2019, No.5). Research staff handling animals were trained in animal care and handling. All efforts were made to minimize animal suffering.

### 2.2. Animals and Cells

Four-to-six-week old female BALB/c mice were purchased from the Research Center for Biomedical Technology (Andreevka, Moscow, RU). Mice were arbitrarily assigned to study groups, had free access to food and sterilized tap water, and were kept on a 12-h light/dark cycle.

*Sf*9 (ATCC CRL-1711) and *Tnms*42 insect cells (a gift from G. Blissard, Boyce Thompson Institute, Ithaca, NY) [27] were routinely propagated in adherent culture in HyClone SFM4 insect cell medium (GE Healthcare, Little Chalfont, UK) at 27 °C and were expanded in suspension culture for recombinant baculovirus working stock generation or recombinant protein expression, respectively.

Madin–Darby Canine Kidney (MDCK) cells (American Type Culture Collection, Manassas, VA, USA) were grown in minimal essential medium (MEM) supplemented with 10% (*v*/*v*) fetal bovine serum (FBS), 5 mM L-glutamine, 25 mM HEPES, 100 U/mL penicillin, 100 μg/mL streptomycin sulfate and 100 μg/mL kanamycin sulfate in a humidified atmosphere of 5% (*v*/*v*) CO_2_.

### 2.3. Infectious Agents

Baculovirus (BV) working stocks were propagated in *Sf*9 cells and titrated by a tissue culture infectious dose 50 (TCID_50_) assay. Briefly, *Sf*9 cells were seeded into sterile, tissue culture-treated 96-well microplates (Corning Inc., Corning, NY, USA) at 32,000 cells/well in a total volume of 100 µL. Baculovirus working stocks or HA-Gag VLP preparations were serially five-fold diluted in medium, including antibiotics (dilution range 2.0 × 10^−1^–4.1 × 10^−9^) in triplicates. Thirty µL of each sample dilution was added to the cells to yield eight replicates per virus dilution. Plates were incubated for five days at 27 °C, and the infection status was assessed on the basis of the expression of an infection reporter, the yellow fluorescent protein (YFP), in infected cells. The viral titer in TCID_50_/mL was calculated using the method of Reed and Muench and was converted into pfu/mL by the Poisson distribution-derived factor 0.69 [28].

Influenza virus A/Puerto Rico/8/1934 (H1N1, termed PR8 hereinafter) was obtained from the Smorodintsev Research Institute of Influenza (St. Petersburg, Russia). NIBRG-121xp, a 6:2 reassortant containing the internal proteins of influenza PR8 and the HA and neuraminidase (NA) proteins from influenza virus A/California/7/2009 (H1N1, termed CAL09 hereinafter) was obtained from the National Institute for Biological Standards and Control (NIBSC, Ridge, UK). Influenza viruses were propagated in nine-day-old embryonated hens’ eggs at 37 °C and titrated by TCID_50_ using MDCK cells and the formula of Reed and Muench.

*Streptococcus pneumoniae* №3405, an invasive human serotype 4 isolate from a patient who died from pneumonia was obtained from the bacterial strain collection of the I. Mechnikov Research Institute for Vaccines and Sera (Moscow, Russia). For bacterial challenge, a freshly inoculated culture was grown to an OD_600_ of 0.6 in tryptic soy medium containing 5% (*v*/*v*) horse blood and was diluted in phosphate-buffered saline (PBS) to a concentration 12.5 × 10^6^ CFU/mL prior to use.

### 2.4. Production and Purification of Influenza HA-Gag VLPs 

Recombinant baculoviruses for the expression of HA-Gag and Gag_only_-VLPs were generated as described in [22]. Briefly, the HA of A/Puerto Rico/8/1934 was expressed under control of the *AcMNPV* p10 promoter, whereas the HIV-1 Gag protein was under control of the *AcMNPV* pH promoter. VLPs were expressed by baculovirus infection of *Tnms*42 cells in Fernbach flasks at a total expression volume of 1200 mL (HA-Gag VLPs) and 600 mL (Gag_only_-VLPs) using a multiplicity of infection (MOI) of 2 and 1, respectively. Expression supernatants were harvested three days post infection and were clarified by two low-speed centrifugation steps (800 g, 10 min, 4 °C; 6000 g, 30 min, 4 °C) using a JLA.9100 rotor (Beckman Coulter, Brea, CA). Clarified supernatants were macrofiltrated in dead-end mode using 0.8 µM syringe filters (Merck Millipore, Burlington, MA). Filtrated supernatants were about 11-fold concentrated and purified from soluble proteins by ultracentrifugation (68,320 g, 2 h, 4 °C, Sw 32 Ti rotor) using a 30% (*w*/*v*) sucrose cushion in 50 mM HEPES pH 7.4. Concentrated and resuspended VLPs were dialyzed for 19 h against a total of 500 dialysis volumes of 50 mM HEPES pH 7.4 using Slide-A-Lyzer^TM^ G2 cassettes with a 10 kDa cutoff (Thermo Fisher, Waltham, MA). Two different treatment methods were employed for live baculovirus inactivation. Dialyzed VLP suspensions were either treated with 5 mM binary ethylenimine (BEI) (Sigma, St. Louis, MO) for 36 h or with 16 mM β-propiolactone (βPL) (Acros Organics, Geel, BE) for 16 h at 37 °C in a water bath. A 0.1 M BEI solution was prepared, as previously described [22]. After inactivation, residual BEI or βPL in the preparation was inactivated by 15 mM or 160 mM sodium thiosulfate (Na_2_S_2_O_3_, final concentration), respectively, and preparations were again dialyzed for 19 h against 500 dialysis volumes of 20 mM HEPES pH 7.4. Samples were stored at 4 °C until further use.

### 2.5. Characterization of HA-Gag VLPs

Total protein content. Total protein content was determined using the Bradford assay in a 96-well microplate format. Briefly, 10 µL of two-fold serial dilutions of the samples were incubated with 200 µL Bradford reagent (Bio-Rad, Hercules, CA, USA) (1:5 diluted in ddH_2_O) in duplicates for five minutes at room temperature (RT) and OD_596_ was measured on a Tecan Infinite M1000 microplate reader (Tecan, Männedorf, CH). Data were fitted to a linear duplicate BSA standard curve (37.5–300 BSA µg/mL).

HA content. The HA content of the HA-Gag VLP preparations was determined using the Influenza A H1N1 (A/Puerto Rico/8/1934) Hemagglutinin/HA ELISA pair set (Sino Biological, Wayne, PA, USA) with some deviations from the manufacturer’s protocol, as previously described [22]. Briefly, we employed a recombinant soluble, trimeric insect cell-expressed PR8 HA protein as calibration standard [26] and pre-treated samples with Zwittergent 3–14 for the solubilization of VLPs.

HA Activity. Hemagglutination activity of the HA-Gag VLP formulations was determined by hemagglutination assay. Briefly, serial two-fold dilutions of the vaccine preparations were prepared in 96-well V-shaped microplates in PBS in a total volume of 50 µL. Fifty µL of chicken red blood cells (cRBCs) isolated from fertilized hens’ eggs, standardized at a cRBC concentration of 4 × 10^7^/mL, were added and plates were incubated at RT for 30 min. The hemagglutination titer (HAU) was defined as the last sample dilution that resulted in complete hemagglutination of cRBCs.

Particle concentration. Particle concentration and size distribution were determined by nanoparticle tracking analysis (NTA) using a NanoSight NS300 (Malvern Instruments, Malvern, UK) equipped with a blue laser module (488 nm), a neutral density filter and a 500 nm fluorescence filter, as described elsewhere [29].

Residual BV infectivity. Five hundred microliter of each preparation (equals 50 βPL_low_/BEI_low_ or 39 Gag_only_ vaccine doses) were inoculated into adherent cultures of *Sf*9 cells in T75 Roux flasks and were incubated for three days at 27 °C. Five hundred microliters of the harvested and clarified supernatants were further added to *Sf*9 cells and incubated for another three days. The absence of viral replication was verified by (1) the lack of YFP expression of inoculated cells, and (2) by the absence of baculovirus-mediated cytopathic effects.

### 2.6. Immunization and Infection Protocol 

Mice were arbitrarily assigned to 14 study groups. No blinding was performed. Immunizations were performed via the intraperitoneal (IP)-route using a total volume of 100 µL experimental vaccine or buffer. Influenza virus and pneumococcus infections were performed intranasally. Before intranasal procedures, mice were lightly anesthetized and were held in an upright position for viral (30 µL /nostril) or bacterial (50 µL/nostril) infection.

**Figure 1:**
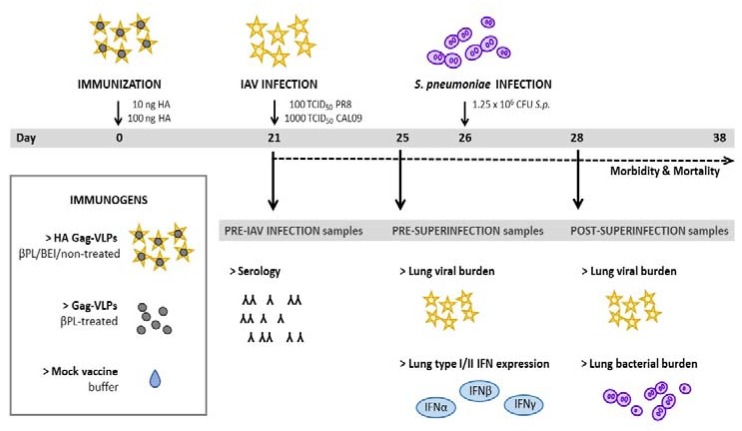
Study design. BALB/c mice were immunized by intraperitoneal injection (IP) with either a βPL/BEI- or non-treated preparation of insect cell-expressed influenza hemagglutinin (HA)-Gag virus-like particles (VLPs), βPL-treated Gag-VLPs or were mock-vaccinated with HEPES buffer. The HA was from PR8. Twenty-one days after immunization, mice were either challenged with 100 TCID_50_ of homologous PR8 or 1000 TCID_50_ of homosubtypic heterologous CAL09 (NIBRG-121xp) and received a secondary infection with 1.25 × 10^6^ CFU of a clinical serotype 4 strain of S. pneumoniae five days later. Immunogenicity of the VLP preparations was assessed in pre-challenge sera three weeks post-immunization. The efficacy of anti-HA immunity in suppressing influenza-mediated innate type I/II interferon (IFN) expression and in limiting exacerbation of disease and mortality (pathogen burden, morbidity, and mortality) was evaluated.

To confirm loss of the BV adjuvant effect by viral inactivation and assess its implications for HA-specific humoral responses, mice (*n* = 10 per group) received a single IP-injection with either BEI-, βPL-, or non-treated HA-Gag VLPs at a dose of 10 ng HA-Gag VLPs (termed BEI_low_, βPL_low_, or live_low_, respectively), βPL-treated Gag-VLPs normalized for the total protein content of HA-Gag VLPs (termed Gag_only_), or were mock-vaccinated with buffer (20 mM HEPES pH 7.4). Five animals were sacrificed for the analysis of HA-specific antibodies in pre-challenge sera (see Section 2.7), while five animals were sacrificed for the assessment of innate immune activation at the vaccination site by analysis of type I/II interferon expression in peritoneal washes (see Section 2.9). To evaluate the efficacy of neutralizing or non-neutralizing influenza HA immunity in the context of secondary pneumococcal infections, animals were immunized and infected as per the study design, given in Figure 1. Four groups of animals were used for the assessment of neutralizing influenza HA-immunity in the context of secondary pneumococcus infections. There, mice were immunized with βPL-or BEI-treated HA-Gag VLPs at a low antigen dose of 10 ng HA (termed βPL_low_, or BEI_low_, respectively, *n* = 26), βPL-treated Gag-only control VLPs normalized for the total protein content of HA-Gag VLPs (termed Gag_only_, *n* = 21) or buffer [termed (−)_vacc_, *n* = 21]. Three weeks later, animals were infected with 100 TCID_50_ of homologous influenza virus PR8 followed by bacterial infection with 1.25 × 10^6^ CFU *S. pneumoniae* five days later. Six groups of animals were employed for the assessment of non-neutralizing influenza HA-immunity after influenza-pneumococcal superinfections. Among this set of mice, four groups of 25–26 animals received βPL- or BEI-treated HA-Gag VLPs at a low (10 ng HA, termed βPL_low_, or BEI_low_) or a high antigen dose (100 ng HA, termed βPL_high_, or BEI_high_). Control groups received βPL-treated Gag-only VLPs (termed Gag_only_, *n* = 21) normalized for the total protein content of to the low dose groups or were mock-immunized with buffer [termed (−)_vacc_, *n* = 24]. Twenty-one days post immunization mice were challenged with 1000 TCID_50_ of a heterologous influenza H1N1 virus (CAL09) followed by bacterial superinfection with 1.25 × 10^6^ CFU *S. pneumoniae* five days later.

To verify the lethal synergism of sequential influenza-pneumococcus infection, additional control groups were mock-immunized and were challenged with a single pathogen on the respective days of infection. Briefly, virus-only groups were infected with 100 TCID_50_ PR8 (termed PR8-only, *n* = 18) or 1000 TCID_50_ CAL09 (termed CAL09-only, *n* = 19) and were mock-infected with buffer five days later, while the bacterium-only group (termed *S.p.-*only, *n* = 14) was mock-infected on day 21 and received 1.25 × 10^6^ CFU *S. pneumoniae* on day 26. Mice were monitored for morbidity and mortality for 17 days after influenza virus infection. Weight was measured daily for five consecutive days post viral infection and was then measured every other day for the rest of the study period. Animals that lost 30% or more of their initial body weight were scored dead and humanely euthanized.

### 2.7. Serology

Twenty-one days post-immunization five mice per group were humanely euthanized for the determination of hemagglutination-inhibition titers (HI titers) in pre-challenge sera. Briefly, individual sera were treated with receptor-destroying enzymes (RDEs) (Denka Seiken, Tokyo, Japan) and were serially two-fold diluted with PBS in 96-well microplates before being mixed with influenza PR8 or CAL09, standardized at 8 HAU. Mixtures were incubated for 60 min at RT and then mixed with a solution of 0.5% (*v*/*v*) chicken RBCs in PBS. After incubation for 60 min at RT, hemagglutination was assessed. The HI titer was calculated from the reciprocal of the highest dilution that completely inhibited hemagglutination of red blood cells.

For the analysis of HA-specific total immunoglobulin G (IgG) titers and IgG isotype profiles, ninety-six-well flat-bottomed Nunc MaxiSorp immunoplates (Thermo Fisher, Waltham, MA, USA) were coated with recombinant insect-cell expressed soluble HA from PR8 containing a trimerization domain [30] at a concentration of 2 µg/mL in PBS at 4 °C overnight. The following day, plates were washed three times with 300 µL PBS-Tween (PBS-T) and wells were blocked by the addition of 300 µL PBS-T + 3% (*w*/*v*) dry-fat milk powder for one hour at room temperature. Serum samples were serially 1:2-diluted in PBS-T with a starting dilution of 1:100 and 100 µL of each dilution was incubated with the coated antigen for two hours at room temperature. After washing three times with PBS-T, 50 µL of either a horseradish peroxidase (HRP)-labeled goat anti-mouse IgG (#A2304, Sigma, St. Louis, MI) or goat anti-mouse IgG1 (#ab97240), IgG2a (#ab97245) or IgG2b secondary antibody (#ab97240, all Abcam, Cambridge, UK) was added at a dilution of 1:3000 in blocking buffer. After incubation for one hour at room temperature plates were again washed three times and were developed with 100 µL SIGMAFAST^TM^ OPD substrate (Sigma, St. Louis, MI, USA) and stopped with 25 µL 3 M H_2_SO_4_. The optical density was measured at 490 nm using a Tecan Infinite M1000 microplate reader (Tecan, Männedorf, Switzerland) and was normalized to blank.

### 2.8. Analysis of Lung Pathogen Burden

On day four afterviral infection four mice from all but the bacterial control group were euthanized by cervical dislocation for the titration of virus loads. Two days after bacterial superinfection (seven days after viral infection) viral and bacterial titers were determined in five mice per group from all study groups. Lungs were removed, thoroughly rinsed with sterile PBS to remove cellular debris and red blood cells and were homogenized and resuspended in one mL of cold PBS. For the titration of bacterial loads, 10-fold dilutions of lung homogenates were titrated on tryptic soy agar plates supplemented with 3% (*v*/*v*) sheep erythrocytes and *S. pneumoniae* colonies were counted. For virus titration, lung homogenates were centrifuged to remove cellular debris (2000 g, 10 min) and 0.1 mL of the clarified lung supernatants were injected into the allantoic cavity of nine-day-old embryonated hens’ eggs to determine the 50% egg infective dose (EID_50_).

### 2.9. Analysis of type I/II Interferon Expression

Type I/II interferon induction was evaluated (1) in peritoneal washes of mice (*n* = 5) receiving either βPL/BEI- or non-inactivated HA-Gag VLPs at a dose of 10 ng HA, βPL-treated Gag-VLPs, or buffer six hours post-immunization and (2) in lung homogenates of immunized animals four days post-viral infection (*n* = 4–5). Briefly, for harvesting IP-washes, mice were sacrificed by cervical dislocation, and one mL of cold PBS was injected into the abdominal cavity using a syringe. Peritoneal washes were collected and clarified by centrifugation (3.500 rpm, 5 min). Lung homogenates were harvested as described in 2.8. IP-washes and lung homogenates were evaluated for the expression of IFNα/β and IFNγ using a LEGENDplex mouse type I/II interferon bead-based multiplex assay (Biolegend, San Diego, CA) according to the manufacturer’s instructions. Assay Buffer and beads were added to samples to reach a final volume of 75 µL per well. Each sample and standard were run in duplicates. The samples were incubated in the dark on a plate shaker (750 rpm) for two hours at room temperature. After washing with 200 µL of Wash Buffer (centrifugation at 1100 rpm for 5 min at 25 °C) samples were incubated with 25 µL of the provided detection antibodies for one hour on a plate shaker. Subsequently, Streptavidin and R-phycoerythrin conjugates were added, and the samples were incubated for an additional 30 min. Following centrifugation, the supernatants were discarded, samples were washed with 200 µL of Wash Buffer, and analyzed using a CytoFlex Flow Cytometer (Beckman Coulter, Brea, CA, USA), and data were analyzed using the LEGENDplex Software, Version 8.0 (BioLegend, San Diego, CA, USA).

### 2.10. Statistical Analyses 

All statistical analyses were performed with GraphPad Prism Version 8.1.0 (GraphPad Software, San Diego, CA, USA). Raw data were assessed for normality of distribution and homogeneity of variances using the D’Agostino–Pearson omnibus test and Brown–Forsythe test respectively before undergoing statistical procedures. Data that did not meet the requirements for parametric statistical tests were log-transformed before analysis. Statistical significance of differences in HI titers between groups was analyzed using One-Way analysis of variance (ANOVA) with the Tukey posthoc test correcting for multiple comparisons on the basis of log-transformed HI titers. Differences in viral titers between groups and between days were analyzed on the basis of log-transformed data using Two-Way ANOVA and a Tukey posthoc test or using Two-Way ANOVA and a Sidak correction factor, respectively. Log-transformed bacterial titers were analyzed by One-Way ANOVA and the Tukey posthoc test or a Kruskal–Wallis test with the Dunn correction factor (in case of non-normality of data). Values below the lower limit of detection (LLOD) were assigned a value of LLOD/2 for statistical analyses. The statistical significance of differences in cytokine concentrations were analyzed on log-transformed data by One-Way ANOVA with a Tukey correction factor or by Welch ANOVA followed by a Dunnett’s T3 multiple comparison test (in case of variance inequality). The LLOD was defined strictly as the lowest analyte concentration of the standard curve, and extrapolated values below were considered as non-detectable. Bodyweight curves were analyzed by multiple t-tests with the assumption of similar standard deviations, and statistical significance was determined using the Holm–Sidak method. Mouse survival was analyzed by the Log-Rank (Mantel–Cox) test.

## 3. Results

### 3.1. Study Design and Murine Superinfection Model

We aimed to assess the distinct role of neutralizing and non-neutralizing immunity towards the influenza HA in preventing post-influenza pneumococcal disease and mortality in a murine BALB/c model. The HI assay was employed as a validated surrogate assay for the measurement of neutralizing anti-influenza immunity. We used a virus-like particle (VLP)-based immunogen due to several reasons: (1) VLPs enabled us to investigate immunity conferred by a single influenza antigen and (2) VLPs allow for the presentation of the HA in its native conformation in particulate and highly immunogenic form [31,32]. We inactivated BV impurities in our preparations with alkylating agents (βPL or BEI) with the aim of abolishing BV-mediated adjuvant effects. Mice were immunized by IP-injection of HA-Gag VLP preparations at different dose levels (termed βPL_low/high_, BEI_low/high_, live_low_), control VLPs (termed Gag_only_) or were mock-vaccinated with buffer [termed (−)_vacc_]. Low antigen doses corresponded to 10 ng of HA, while high doses were equal to 100 ng of HA. The intraperitoneal route of immunization was chosen due to its technical convenience and because it ensures fast drainage of antigen-presenting cells (APCs) to local lymph nodes and thereby results in robust B- and T-cell responses [33]. Twenty-one days post-immunization mice were intranasally infected with either homologous HA-matched influenza PR8 or a heterologous, non-matched influenza virus of the same subtype (CAL09). Previous studies in this field revealed that *S. pneumoniae* is highly lethal when administered within five to seven days post influenza infection [12,34,35]. Therefore, the sequential viral-bacterial-infection model was set up accordingly, and animals were challenged with bacteria five days post-influenza infection and samples were collected, as indicated in Figure 1.

To characterize the two employed superinfection models we performed mono- or dual-infections with viruses and bacteria as outlined in Figure 1**.** Mice mono-infected with a viral dose of 100 TCID_50_ of influenza PR8 or 1000 TCID_50_ of CAL09 demonstrated mean viral pulmonary day four titers of 7.5 and 5.8 log_10_ EID_50_/mL, respectively, which dropped significantly on day 7 (Figure S1A). In stark contrast, bacterial superinfections lead to a rebound in viral titers on day 7 (both p_adj_ < 0.0001). At the same time, influenza-infected mice displayed significantly higher pulmonary bacterial loads than mice without a history of influenza (Figure S1B). While the pneumococcal mono-infection was non-lethal in mice, antecedent infection with influenza PR8 or CAL09 dramatically enhanced severity of the disease leading to 100% mortality within two or eight days post bacterial infection, respectively (Figure S1C,D).

### 3.2. Effect of Baculovirus Inactivation on Influenza VLP Immunogenicity

Purified insect cell-derived enveloped VLPs may contain bioprocess-derived, co-purified baculovirus impurities that are commonly inactivated with alkylating agents, such as βPL or BEI, for *in vivo* studies [36,37]. βPL is routinely employed for the manufacturing of inactivated influenza vaccines but was associated with amino acid modifications that affect HA function and antigenicity [36,38,39,40]. BEI is suggested to preserve protein conformation and antigenicity better [40], but has been less frequently used for the preparation of experimental influenza vaccines. We, therefore, aimed to compare how these two inactivation strategies influence HA function and HA-specific humoral immunity and protective efficacy.

**Figure 2:**
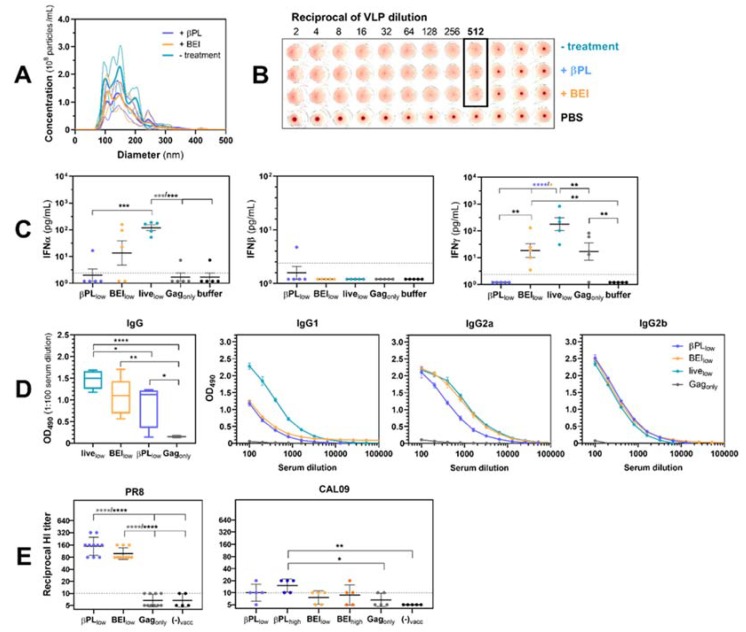
Effect of baculovirus inactivation on influenza VLP vaccine immunogenicity. (**A**) Preservation of the particulate integrity and (**B**) HA activity of the VLP formulations after treatment with 16 mM βPL or 5 mM BEI for 16h and 36h at 37 °C respectively was verified by dynamic light scattering analysis and hemagglutination assay, respectively. Broad and narrow lines in panel (A) give the mean and SD of the measured particle concentrations, respectively. (**C**) Abolishment of vaccine vector-induced local innate immune activation reflected by IFNα, IFNβ and IFNγ expression was evaluated six hours post-IP-immunization with the respective VLP preparations or buffer in peritoneal washes of mice (*n* = 5 per group). Data represent log-transformed results for individual animals and group means ± SEM. (**D**) The effect of baculovirus (BV)inactivation on the magnitude and quality of the PR8 HA-specific humoral response was assessed in mouse serum samples taken three weeks post-immunization. Total IgG titers were determined from individual sera (*n* = 5 per group), IgG1, IgG2a, and IgG2b isotype levels were determined in pooled sera in duplicates. (**E**) HI antibodies against the vaccine-matched influenza PR8 or the non-matched CAL09 challenge strain were assessed in pre-challenge sera (*n* = 4–10). Color code vaccine groups: βPL_low_ (light blue), βPL_high_ (dark blue), BEI_low_ (light orange), BEI_high_ (dark orange), live_low_ (turquoise), Gag_only_ (grey), Non-vaccinated (black). Symbols represent HI titers of individual mice, lines and error bars indicate the group geometric mean titer and SD. Dashed lines indicate the lower limit of detection (LLOD) at a HI titer of 1:10. Statistical significance of differences in mean IFN titers, IgG titers, and HI titers (log-transformed data) were analyzed by One-Way ANOVA and a Tukey correction factor with * *p_adj_* < 0.05, ** *p_adj_* < 0.01, *** *p_adj_* < 0.001, and **** *p_adj_* < 0.0001.

Neither of the employed inactivation protocols affected the structural integrity of VLPs (Figure 2A) or the hemagglutination capacity of the HA (Figure 2B). The latter of which suggests that antigenic sites close to the receptor-binding domains have been functionally preserved. Five hundred microliter of the undiluted inactivated VLP preparations (equal to 50 low antigen doses) were inoculated in cell culture flasks containing *Sf9* cells and resulted in no detectable expression of the infection reporter YFP (data not shown). Baculoviruses are potent inducers of innate immunity as they trigger type I/II IFN expression and recruitment of immune cells to the site of immunization [41,42,43]. We aimed to confirm the elimination of the BV adjuvant effect after chemical treatment and assessed interferon responses at the injection site six hours post-immunization (Figure 2C). In accordance with previous studies, we observed elevated IFNα and IFNγ expression in peritoneal washes of mice immunized with a non-treated HA-Gag VLP formulation containing 1.7 × 10^5^ pfu infectious BVs [41,44]. BV inactivation with βPL eliminated IFNα (*p* = 0.0006) and IFNγ induction (*p* < 0.0001) at the site of vaccination. Similarly, IFNα and IFNγ expression in mice immunized with BEI-treated HA-Gag VLPs or βPL-treated Gag_only_ control VLPs was successfully reduced [IFNα: *p_adj_* = 0.0004 (Gag_only_), IFNγ: *p_adj_* = 0.0297 (BEI_low_), *p_adj_* = 0.0219 (Gag_only_)], but was not completely abolished. None of the groups demonstrated IFNβ expression in peritoneal washes six hours post-immunization.

Due to their adjuvant properties, infectious baculoviruses were shown to enhance humoral and cellular responses against co-administered antigens in mice [36,43,44]. We sought to assess the implication of BV inactivation on the magnitude and quality of humoral responses in pre-challenge sera collected 21 days post-immunization (Figure 2D). Serum IgG titers were highest after immunization with formulations capable of IFN-induction. βPL-mediated BV inactivation was accompanied by the greatest reduction in total serum IgG concentration (*p_adj_* = 0.0492). Immunization with a formulation containing live baculovirus elicited a balanced IgG1:IgG2a:IgG2b antibody response. The immunoglobulin subclass profile elicited by both inactivated HA-Gag VLP preparations was characterized by a reduction in IgG1 antibodies, indicating a shift towards a Th1 immune response. To test the functionality of the antibodies, pre-challenge serum samples were tested for the induction of HI antibodies (neutralizing antibodies) for the respective challenge strains (Figure 2E). Mice immunized with βPL-treated and BEI-treated HA-Gag VLPs at a dose of 10 ng HA displayed comparable HI titers against PR8 (βPL_low_: GMT 149.3; BEI_low_: 98.5, *p* = 0.1213)**.** Only mice immunized with βPL_high_ consistently demonstrated detectably, but low, HI titers against CAL09 (GMT, 15.6, *p* = 0.007).

### 3.3. Effective Anti-HA Immunity Suppresses Influenza-Mediated IFN Type I/II Responses in the Lungs of Infected Mice

**Figure 3:**
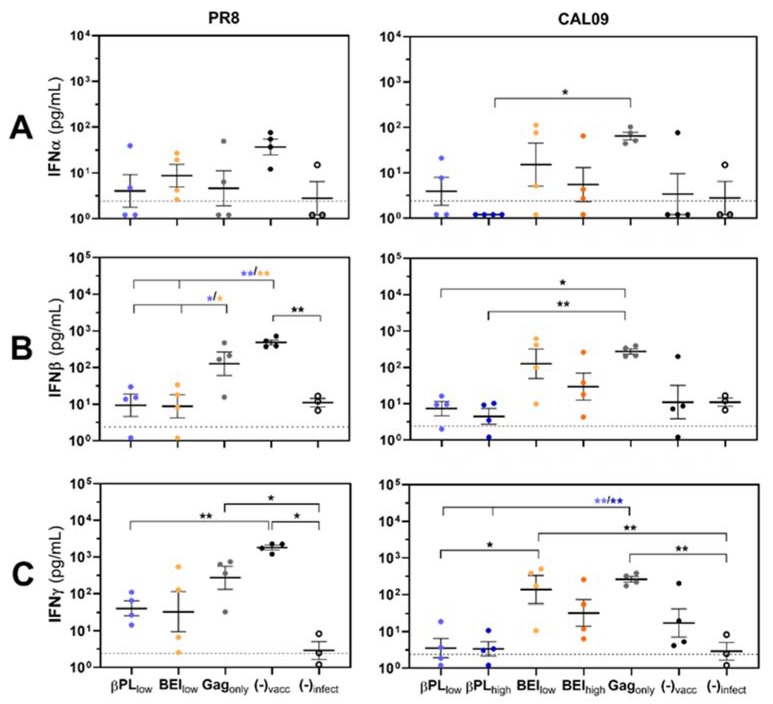
Effective anti-HA immunity suppresses influenza-mediated IFN type I/II responses in the lungs of infected mice. Lung homogenates from immunized or mock-vaccinated mice (*n* = 4 per group) infected with vaccine-matched PR8 (left panel) or non-matched CAL09 (right panel) were analyzed for the expression of (**A**) IFNα, (**B**) IFNβ, and (**C**) IFNγ four days post-viral infection. Basal type I/II IFN expression levels were analyzed in naïve, non-infected mice (*n* = 3). Symbols represent the mean IFN concentration of a duplicate measurement from a single mouse and lines and error bars indicate group means +/− SEM. Color code vaccine groups: βPL_low_ (light blue), βPL_high_ (dark blue), BEI_low_ (light orange), BEI_high_ (dark orange), Gag_only_ (grey), non-vaccinated (black, full circle), naïve (black, open circle). Statistical significance of differences in mean IFN-concentrations between groups was analyzed by One-Way ANOVA and a Tukey posthoc test or by Welch ANOVA followed by a Dunnett‘s T3 posthoc test for multiple comparisons (**C**, left panel) on basis of log-transformed data. The dashed line indicates the defined LLOD and represents the lowest analyte concentration of the calibration curve (2.4 pg/mL). Asterisks indicate the significance level giving adjusted *p*-values for multiple comparisons with * *p_adj_* < 0.05 and ** *p_adj_* < 0.01.

Interferon type I/II expression in response to an influenza virus infection is a host factor suggested to increase the susceptibility of an individual to secondary bacterial infection [5,6,7,8,14,45,46]. IFN signaling was associated with the negative regulation or depletion of innate immune cells critical for early bacterial control [5,7,11,12,34]. In an effort to evaluate whether neutralizing and non-neutralizing anti-HA immunity can prevent this host sensitization, we assessed pulmonary concentrations of IFNα, IFNβ and IFNγ one day before bacterial challenge (Figure 3A–C).

Non-vaccinated, PR8-infected animals displayed significantly higher pulmonary IFNβ (1.6-log increase, *p_adj_* = 0.0066) and IFNγ concentrations (2.8-log increase, *p_adj_* = 0.0305) than naïve mice (Figure 3B,C, left panels). Immunization with HA-matched VLP preparations, irrespective of the BV inactivation method, could dampen influenza-induced cytokine expression to concentrations observed in naïve mice (Figure 3C, left panel). IFNβ and IFNγ expression levels in the lungs of Gag_only_-immunized and PR8-infected animals were similar to non-vaccinated mice (Figure 3C, left panel). To our surprise, non-vaccinated, CAL09-infected mice demonstrated no increase in lung type I/II IFN expression in comparison to naïve, non-infected mice (Figure 3, right panels). We also observed no differences in type I/II IFN expression between immunized and non-immunized, infected animals. Interestingly, the group of mice immunized with Gag_only_-VLPs exhibited the most homogenous IFN response pattern among all analyzed groups and displayed significantly elevated IFNγ expression compared to naïve mice (2-log higher, *p* = 0.0445) (Figure 3C, right panel). In general, there was a trend for higher type I/II IFN expression in the lungs of animals immunized with BEI-treated HA-Gag preparations or Gag_only_-VLPs, while βPL-treated HA-Gag immunogens were associated with the lowest induction of interferons after CAL09 infection.

### 3.4. Neutralizing and Non-Neutralizing Anti-HA Immunity Restrain Viral Replication and Viral Rebound After Secondary Pneumococcus Infection

**Figure 4:**
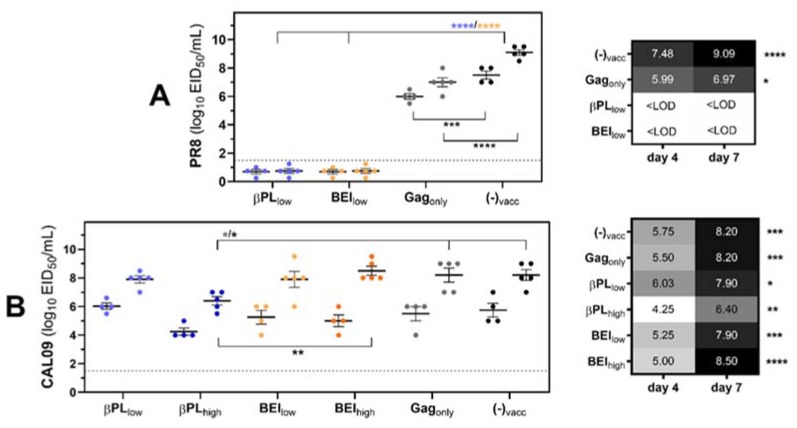
Neutralizing and non-neutralizing anti-influenza HA immunity restrains viral replication and viral rebound after secondary pneumococcus infection. Mice received one intraperitoneal dose of the respective preparations or buffer, and on day 21 were intranasally infected with influenza followed by S. pneumoniae challenge five days later. Viral pulmonary loads were analyzed in mice (*n* = 4–5 per group) infected with (**A**) 100 TCID_50_ of homologous influenza PR8 or (**B**) 1000 TCID_50_ of homosubtypic heterologous CAL09. Figures give viral day four titers (one day before bacterial challenge) juxtaposed with day seven titers (two days post bacterial challenge) for each vaccine group. Color code vaccine groups: βPL_low_ (light blue), βPL_high_ (dark blue), BEI_low_ (light orange), BEI_high_ (dark orange), Gag_only_ (grey), non-vaccinated (black). Symbols represent the titer of an individual mouse and lines and error bars indicate group mean titers +/− SEM. Heatmaps display group mean viral pulmonary titers on day four and day seven. The dashed line at 1.5 log_10_ EID_50_/mL indicates the LLOD. Viral titers were log-transformed and differences between groups and between days were analyzed by Two-Way ANOVA followed by a Tukey posthoc test or a Sidak posthoc test, respectively, with * *p_adj_* < 0.05, ** *p_adj_* < 0.01, *** *p_adj_* < 0.001, and **** *p_adj_* < 0.0001.

We next studied the effect of vaccine-matched (neutralizing) and vaccine-mismatched (non-neutralizing) anti-HA immunity on viral replication in the lungs one day before and two days after bacterial superinfection. Non-vaccinated mice displayed high viral pulmonary titers on day four (mean titer +/− SEM: 7.5 +/− 0.288 log_10_ EID_50_/mL) and titers increased about 400-fold (*p_adj_* < 0.0001) after bacterial secondary infection (Figure 4A).

Immunization with βPL_low_ or BEI_low_ could fully restrict PR8 replication on day four post-infection (*p_adj_* < 0.0001). Interestingly, despite the absence of an influenza antigen, Gag_only_-immunized mice displayed reduced pulmonary PR8 titers on day four (*p_adj_* = 0.0027) and seven (*p_adj_* < 0.0001) and a lower rebound in viral titers after bacterial superinfection (Figure 4A). In contrast, PR8 HA-specific immunity did not restrain CAL09 virus replication on day four (range: 4.3–6.0 log_10_ EID_50_/mL) or lower the rebound in viral titers after *S. pneumoniae* superinfection (Figure 4B). Yet, we observed a reduction in CAL09 day seven titers in mice immunized with βPL_high_. There, the viral load after the secondary infection was 1.8-log (*p_adj_* = 0.0163) or 2.1-log (*p_adj_* = 0.0033) lower than in non-vaccinated or BEI_high_-immunized animals, respectively.

### 3.5. Neutralizing Anti-Influenza HA Immunity Renders Mice Capable of Controlling a Secondary Pneumococcal Infection 

**Figure 5:**
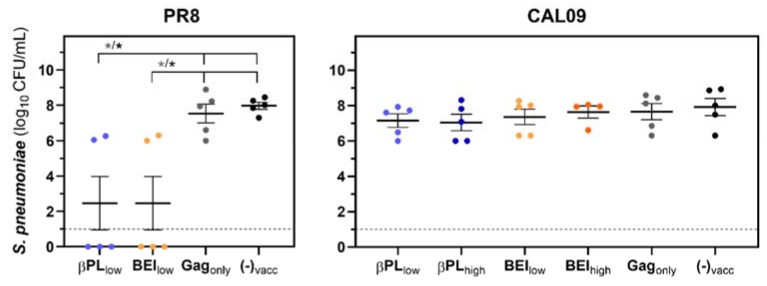
Neutralizing anti-influenza HA immunity renders mice capable of controlling a secondary pneumococcal infection. Immunized or mock-immunized and PR8-infected (left panel) or CAL09-infected mice (right panel) were challenged with 1.25 × 10^6^ CFU of a clinical serotype 4 strain of S. pneumoniae five days post influenza and the bacterial burden was assessed in the lungs of mice two days after bacterial infection (*n* = 3–5 per group). Color code vaccine groups: βPL_low_ (light blue), βPL_high_ (dark blue), BEI_low_ (light orange), BEI_high_ (dark orange), Gag_only_ (grey), non-vaccinated (black). Symbols represent the titer of an individual mouse and lines and error bars indicate group means +/− SEM. The dashed line indicates the LLOD of the assay. Statistical significance of mean titers between groups was analyzed by One-Way ANOVA and the Tukey posthoc test or a Kruskal–Wallis test with a Dunn’s correction factor on basis of log-transformed data with * *p_adj_* < 0.05.

We confirmed that influenza virus infection with both viral strains suppressed antibacterial control (Figure S1B) and we, therefore, investigated whether homologous, neutralizing and heterologous, non-neutralizing anti-HA immunity can revert this effect. Bacterial growth was evaluated in the lungs of immunized and influenza-infected animals two days after secondary bacterial infection (Figure 5).

Neutralizing anti-HA immunity was associated with a significant reduction (−5.5 log CFU/mL, *p_adj_* = 0.0132) of the mean bacterial density in the lungs of immunized mice in contrast to non-vaccinated animals (Figure 5, left panel). In three out of five mice, pneumococcus was completely cleared from the lungs. Two mice exhibited bacterial loads similar to what we observed in animals mono-infected with *S. pneumoniae* (Figure S1B). Interestingly, although Gag_only_-immunized mice were able to dampen PR8 virus replication in the lungs (Figure 4A), they were not able to lower the pneumococcal burden two days after bacterial challenge (*p_adj_* = 0.992). We observed no reduction in bacterial pulmonary titers in mice immunized with mismatched HA-Gag VLP preparations (Figure 5, right panel).

### 3.6. Influenza-Specific and Non-Specific Immunity Provide a Survival Advantage After Secondary Pneumococcal Infection 

Influenza vaccination with IIVs and LAIVs has proven beneficial for preventing lower respiratory tract complications and death from secondary pneumococcal infections in murine models [20,21]. Yet, no such study assessed the impact of neutralizing or non-neutralizing anti-HA immunity alone in a lethal *in vivo* influenza-pneumococcus infection model.

**Figure 6:**
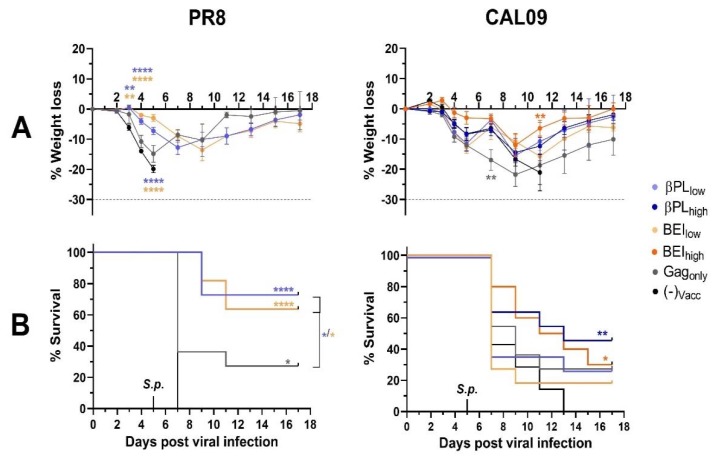
Influenza-specific and non-specific immunity provides a survival advantage after secondary pneumococcal infection. Mice received one intraperitoneal dose of HA-Gag VLPs at low HA antigen dose (10 ng), control VLPs or buffer or (**C**) VLPs at a high HA antigen dose (100 ng) or buffer. Three weeks later, mice were infected with homologous influenza PR8 or homosubtypic heterologous CAL09 followed by bacterial challenge with S. pneumoniae five days post influenza infection. Weight loss (**A**) and survival (**B**) was monitored over a period of 17 days post-viral infection. Animals that lost 30% of their initial body weight (dashed line) were scored dead and humanely euthanized. Data represent the mean weight of each group +/− SEM. Weight loss curves were analyzed with multiple t-tests using the Holm–Sidak correction factor for multiple comparisons, and survival curves were analyzed with the Log-Rank test, with * *p_adj_* < 0.05, ** *p_adj_* < 0.01 and **** *p_adj_* < 0.0001.

Immunization with βPL_low_ and BEI_low_ protected mice from morbidity associated with homologous influenza virus infection (Figure 6A, left panel). In these groups weight loss was significantly lower than in non-vaccinated animals within the first five days after viral infection [(−)_vacc_: −19.9%, βPL: −7.2%, BEI: −3%; both *p_adj_* < 0.0001)]. After secondary infection, all non-vaccinated mice (11/11) reached the clinical endpoint for humane euthanasia within two days after bacterial inoculation (median survival time: seven days). In contrast, matching influenza HA-immunity elicited by βPL_low_, and BEI_low_ prevented mortality in eight (73%) and seven (64%) out of 11 animals, respectively (both *p* < 0.0001) (Figure 6B, left panel). Interestingly, also immunization with control-VLPs (Gag_only_) conferred partial protection from mortality (three out of 11 mice, 27.7%; *p* = 0.0308), but did not prolong median survival time. Sequential infection with CAL09 + *S. pneumoniae* was 100% lethal for non-vaccinated mice with a median survival time of seven days, but with slightly slower mortality kinetics compared to infection with PR8 + *S. pneumoniae* (Figure 6B). In this setting, only βPL_high_-, and BEI_high_-immunization conferred significant protection from mortality (5/11 mice, *p* = 0.0093 and 4/11 mice, *p* = 0.014, respectively) and prolonged median survival to 13 and 12 days, respectively. We observed no significant protection from mortality associated with sequential CAL09-pneumococcus infection in animals immunized with Gag_only_ control VLPs (*p* = 0.1778).

## 4. Discussion

The link between influenza and secondary bacterial infection has been recognized for many years. There is a large body of research showing that influenza-mediated physical and physiological changes of the host leave an individual more susceptible to bacterial superinfection [5,6,8,12,46,47]. Yet, influenza vaccine-induced immunity is almost exclusively evaluated using protection from disease or mortality after an influenza infection as the study endpoint. The potential of influenza vaccination to abrogate the lethal viral-bacterial synergism remains a little-investigated field. In the present study, we demonstrated that neutralizing influenza HA head-directed immunity conferred by different HA-Gag VLP formulations allowed BALB/c mice to control a secondary pneumococcal infection better and reduced mortality from 100% to 27% or 36%, respectively (both *p* < 0.0001). This is impressive, considering the low non-adjuvanted antigen dose of 10 ng HA and the single immunization regimen we employed. It is, however, also indicative of the multifaceted interplay between viral, host, and bacterial factors that have to be considered, since 30–40% of mice could not be protected from mortality despite (1) high HI antibody titers (2) effective control of viral replication, and (3) successful reduction of the bacterial lung burden.

Interferon induction is an innate immune response to viral infection and type I/II IFNs are collectively acknowledged for their antiviral and immune-stimulatory activities [48,49]. Nevertheless, there is increasing evidence of the detrimental effects of interferon signaling in the context of SBIs post-influenza [5,7,34]. We, therefore, assessed interferon induction in the lungs of immunized and influenza-infected mice before secondary bacterial infection with the aim to relate the degree of innate immune induction to the capability of mice to control bacterial outgrowth. Experimental studies indicate that bacterial infections coinciding with a narrow time window of influenza-induced IFN expression result in unrestricted bacterial growth with severe consequences [7,34,35]. Indeed, mice deficient in type I IFN signaling (IFNR^−^/^−^ knock-out mice) could adequately produce chemoattractants that guide neutrophil recruitment to the site of infection. This eventually resulted in improved secondary bacterial clearance and survival in contrast to wild type mice [5,34,46]. Similarly, pulmonary IFNγ abundance induced by influenza virus infection can be mimicked by direct IFNγ inoculation and was shown to suppress pulmonary antibacterial defense mechanisms. This phenotype, however, could be prevented by antibody-mediated IFNγ neutralization after influenza infection [7]. In our study, anti-HA immunity conferred by two differentially treated HA-Gag VLP preparations restrained homologous viral replication by day four post-infection and thereby dampened innate type I/II IFN expression in the lungs. In this setting, 60% of mice were capable of fully controlling bacterial outgrowth two days after bacterial infection. We assume that neutralizing anti-HA immunity could counteract virus-mediated IFN responses of the host and thereby rescue its phagocytic and bactericidal capacity to rapidly clear the pneumococcal challenge dose.

Considering the incomplete protection provided by neutralizing anti-HA immunity we were surprised that non-influenza-specific immunity conferred by immunization with Gag_only_ VLPs was associated with a significant reduction in viral titers and protection from mortality (27%, *p* = 0.0308) in the absence of suppressing bacterial replication. It is of note that upon inoculation of a volume equal to 39 Gag_only_ vaccine doses into an adherent *Sf9* cell culture, we noticed that BV had not been inactivated to the completeness, as we observed a few cells expressing the YFP infection reporter (data not shown). This was in contrast to what we observed for HA-Gag formulations. Gag_only_-immunization, therefore, resulted in the activation of innate immunity, such as IFNγ expression at the site of immunization. Interestingly, these mice also exhibited significantly elevated pulmonary IFNγ concentrations after infection with PR8 or CAL09. Infectious baculoviruses possess strong adjuvant activities due to the presence of abundant unmethylated CpG motifs throughout their viral genome [50]. Unmethylated CpG motifs are sensed by mammalian Toll-like receptor 9 (TLR9), a protein component of the innate immune system expressed in many immune cells. Infectious baculovirus was demonstrated to activate murine NK cells either directly via TLR9 [51] or indirectly via cytokine signaling of transduced murine DC cells [42]. NK cells that had been previously activated by cytokines were shown to have a cell-intrinsic enhanced capacity to respond to secondary stimuli and produce more IFNγ upon restimulation [52]. We speculate that innate immune training by live baculovirus present in the Gag_only_-VLP preparation led to a higher production of IFNγ after restimulation with influenza virus. Thereby, the virus may have encountered an immunologically primed environment with higher numbers of activated macrophages, neutrophils, and natural killer cells. Some of these cells are professional antigen-presenting cells, and likely may have had superior antigen-presenting properties upon restimulation with influenza virus. The phenomenon of trained immunity was also observed by Norton and colleagues in BALB/c mice that received CpG oligodeoxynucleotides one day before being challenged with a lethal dose of influenza PR8 [53]. CpG administration induced a local inflammatory response that resulted in a significant reduction in morbidity and mortality upon influenza infection. In our study, the time span between vaccine-induced innate immune activation and re-activation by virus encounter is much longer (3 weeks). In this respected, it would have been of interest to assess the duration of the primed functional state elicited by residual baculovirus in the preparation. These are important results, as innate immune training may open a door for novel vaccine formulations that unlike conventional vaccines, which focus on the elicitation of a specific response to the nominal pathogen, non-specifically stimulate immunity against a wider array of pathogens.

Treatment of the VLP preparations with alkylating agents, such as βPL or BEI, results in the alkylation of nucleotides other than thymidine or uracil and induces DNA nicks or crosslinks DNA strands [54]. β-propiolactone is of widespread industrial use. It is commonly employed for the preparation of IIVs and for baculovirus inactivation in preparations intended for preclinical use [36,55]. In contrast to previous beliefs, studies demonstrated βPL to react with several amino acids (most notably with cysteine, methionine and histidine residues) [55] and to alter the function of key viral antigens in vaccine preparations [38]. Binary ethylenimine is another alkylating agent employed for baculovirus inactivation but is suggested to preserve antigenic sites of a protein better and was therefore chosen as an alternative strategy for viral inactivation [40]. Inactivation to completeness was confirmed by the absence of viral replication and associated expression of YFP in cells upon inoculation of a volume equal to 50 vaccine doses (βPL_low_/BEI_low_) into *Sf9* cell cultures. Both inactivation protocols could preserve the hemagglutination function of the HA and VLPs were highly immunogenic at single low antigen doses. Both immunogens induced similar titers of HI antibodies and of total HA-specific IgGs. Immunoglobulins displayed an isotype profile indicative of a Th1-polarized response. Our serological analyses suggest that BEI-treatment of experimental influenza vaccines demonstrate a feasible alternative to βPL inactivation.

IgG subclasses differ in their ability to induce antibody-dependent cell-mediated cytotoxicity (ADCC) and to activate the complement pathway. The Fc portion of murine IgG2a antibodies bind to activator Fc receptors and complement components with high affinity and thereby are most potently activating ADCC functions. It is of note that broadly-neutralizing anti-HA stalk antibodies with group 1 and 2 reactivity may mediate protection in an FcγR-dependent manner and are superior inducers of ADCC as compared to anti-HA head-directed antibodies [56]. Anti-stalk antibodies bind to the membrane-proximal part of the HA and do not interfere with binding of the virus to host cells. The HI assay, a surrogate assay for the detection of neutralizing anti-HA antibodies capable of preventing virus attachment to host cells, therefore does not reveal the presence of such antibodies in sera.

In our sequential influenza-pneumococcus infection model using the vaccine non-matched influenza CAL09 challenge strain, we observed 36% and 46% survival in mice immunized with high HA-Gag VLP doses (100 ng HA). Interestingly, this protective effect was observed in the absence or with only minor induction of HI antibodies. However, we observed enhanced viral clearance in βPL_high_-immunized animals on day seven (two days post bacterial challenge). Similarly, in a recent report by Choi et al., vaccination with an alum-adjuvanted matched trivalent influenza vaccine at a dose of 3 µg HA was able to fully protect from lethality after secondary *S. aureus* infection, despite not being able to elicit detectable HI-antibodies in two-thirds of the mice and to reduce viral or bacterial replication [20]. It is tempting to speculate that the protective effect we observed was partially due to the action of broadly-neutralizing anti-stem antibodies with ADCC function.

Another unexpected finding was that mice infected with CAL09 did not demonstrate elevated pulmonary type I/II IFN expression compared to non-infected mice. We observed this effect despite high viral replication (~6 log_10_ EID_50_/mL) in the lungs. Differences in IFNα/β/γ production after influenza infection could be either a consequence of more rapid virus replication leading to a stronger stimulus for IFN expression or may be due to host- or virus-intrinsic factors. We demonstrated slower replication kinetics for CAL09 than for PR8, which is reflected by about 1.8-log lower mean pulmonary titers four days after viral infection. It is possible that this lower viral pulmonary load provided a stimulus that was below the threshold for potent innate immune activation. Evidence that supports this theory comes from our results on IFN expression in one of our PR8-infected control groups (Gag_only_). There, Gag_only_-immunization affected PR8 replication in the lungs and was accompanied by a 1.5-log drop in viral titers on day four to a level similar to what we observed for CAL09 replication. This reduction in viral titers was sufficient to reduce IFNβ expression to basal expression levels observed in naïve, non-infected mice. The influenza NS1 protein is a virulence factor that counteracts the host innate immune response by acting as interferon-antagonist and thereby affects viral replication [57]. CAL09 (NIBRG-121xp) is a reassortant virus with internal proteins donated by PR8. The NS1 proteins of the two employed challenge viruses are therefore assumed to be similar or identical and have the same potential to inhibit interferon induction. In addition to viral ssRNA and dsRNA, carbohydrate moieties that decorate the virus particle were shown to mediate early host detection by C-type lectin binding [58]. In this respect, the abundance and composition of glycans on the influenza glycoproteins HA and NA may provide differential triggers for lectin-recognition and thereby affect subsequent virus aggregation, complement fixation, opsonization, phagocytosis and induction of inflammatory responses [45,59]. Our two challenge viruses not only display HA and NA proteins derived from two H1N1 strains isolated almost 75 years apart they also have a different history of propagation. Protein-intrinsic factors, as well as the propagation history of the viruses thereby, may contribute to the early recognition of the virus via its sugar decoration and affect its innate immune triggering capacity and replication kinetics. These findings suggest that the influenza-induced interferon signature at the time of bacterial infection cannot be used as a single read-out to estimate susceptibility of a host to secondary pneumococcal infection.

A limitation of our study was that we exclusively investigated type I/II IFN expression and did not measure other cytokines that would more specifically let us estimate the progress of secondary bacterial infections. In this respect, γδ T cell-mediated expression of Th17-related cytokines (i.e., IL-17A, IL-23) were demonstrated to be critical for the control of respiratory bacterial infections. Type I IFN expression was shown to affect γδ T-IL-17 secretion in an IFN-dose-dependent manner, which ultimately results in the dysfunction of neutrophil recruitment and activity [9]. Interestingly, also, the influenza HA was recently demonstrated to selectively modulate innate IL-23/IL-12 and adaptive IL-17A responses to secondary pneumococcus infection and thereby constitutes an IFN-independent pathway for the inhibition of the Th17 pathway [14].

## 5. Conclusions

In summary, we studied the potential of neutralizing and non-neutralizing influenza HA-specific immunity in providing off-target protection against post-influenza pneumococcal infections. We evaluated HA-specific immunity in the context of modulating host factors (interferon type I/II expression) associated with its sensitization to secondary infection and superinfection-associated enhancement of pathogen replication, morbidity and mortality in BALB/c mice. Insect cell-expressed VLPs have proven an effective and highly immunogenic vaccine platform to allow us to investigate our research questions at convenient single, low antigen doses of 10–100 ng HA. We tested two different baculovirus inactivation methods and both, βPL and BEI, demonstrated feasible for the inactivation of BVs in experimental influenza VLP vaccines without compromising immunogen quality and antigenicity. We presented indications that type I/II IFN responses during influenza infections may not be fully reliable markers for assessing the risk of secondary bacterial infections post-influenza. In our studies, we unexpectedly observed substantial but incomplete protection despite high HI titers induced by matching vaccine preparations, and at the same time, we demonstrated survival advantages in the absence of pre-challenge serum HI antibodies for a mismatched challenge virus. This suggests that both neutralizing and non-neutralizing immunity towards the HA may have implications in the consequences of post-influenza pneumococcal infections. HI titers, the current surrogate correlates for the prevention of influenza infections may therefore not be reliable indicators for estimating the consequences of post-influenza secondary bacterial infections. In addition, our data suggest that the nature of the vaccine vector and vaccination per se may influence the outcome of this multi-infection scenario by a mechanism termed immune training, which leaves an individual more responsive for pathogen encounters.

## Supplementary Materials

The following are available online at https://www.mdpi.com/2076-393X/7/4/187/s1, Figure S1: Secondary pneumococcus infection after influenza virus infection with PR8 and CAL09 exacerbates disease and mortality in BALB/c mice.
