# Supplementary material for: The Potential of Influenza HA-Specific Immunity in Mitigating Lethality of Postinfluenza Pneumococcal Infections
Source: Vaccines (Basel). 2019 Nov 17;7(4):187. doi: 10.3390/vaccines7040187 (PMC6963476; doi:10.3390/vaccines7040187)
Supplement: Supplementary file 1 [file vaccines-07-00187-s001.pdf]

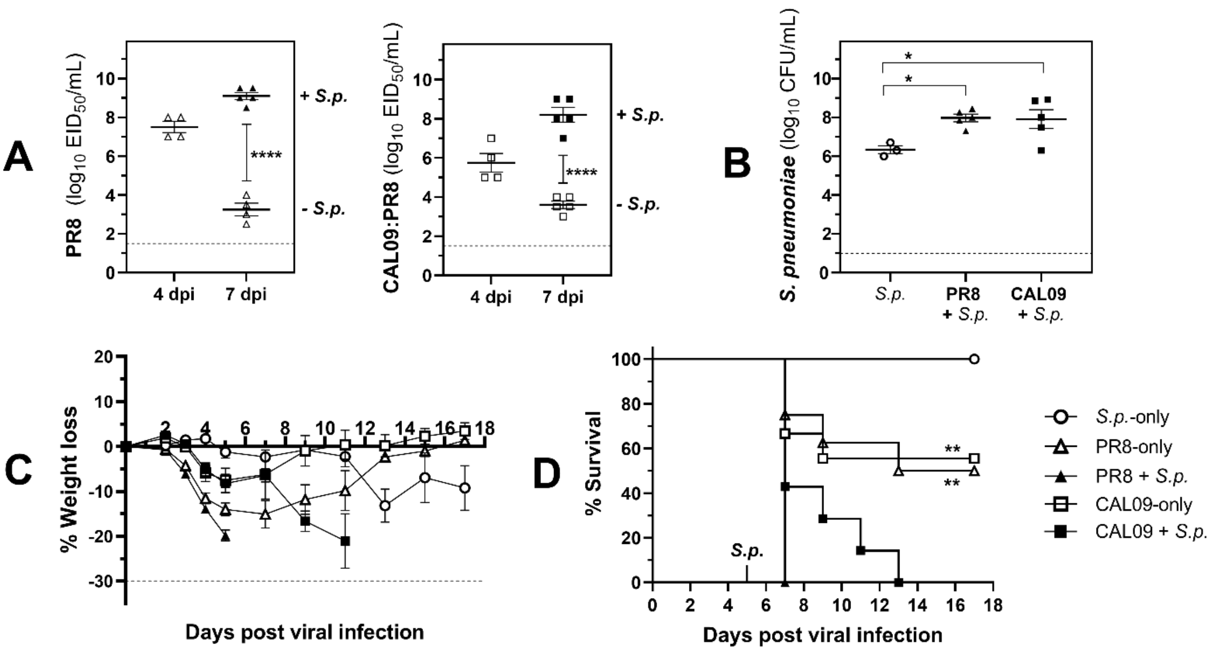

Figure S1. Secondary pneumococcus infection after influenza virus infection with PR8 and CAL09 exacerbates disease and mortality in BALB/c mice. Mice received one intraperitoneal sham-vaccination with buffer and on day 21 were infected with 100 $TCID_{50}$ of influenza PR8 or 1000 $TCID_{50}$ CAL09 followed by secondary bacterial infection with 1.25×10^6 CFU *S. pneumoniae* five days after viral infection. Sham-infected mice received buffer on the respective days. (**A**) Viral pulmonary titers one day before and two days after bacterial infection as well as (**B**) bacterial pulmonary loads two days post bacterial infection was assessed in 3-5 mice per group. Symbols represent the titer of an individual mouse and lines and error bars indicate group mean titers +/- SEM. The dashed line at 1.5 log_{10} EID_{50}/mL and 10 CFU/mL indicate the LLOD of the respective assays. (**C**) Weight loss and (**D**) mortality was monitored over a period of 17 days post viral infection. Animals that lost 30% of their initial body weight (dashed line) were scored dead and humanely euthanized. Data represent the mean weight of each group +/- SEM. Differences in viral pulmonary titers on day seven were analyzed by Two-Way ANOVA with a Tukey post-hoc test and bacterial loads were analyzed by One-Way ANOVA with a Tukey correction factor. Weight loss curves were analyzed by multiple t-tests with a Holm-Sidak correction factor, survival curves were analyzed by a Log-rank test. Asterisks indicate the significance level with * $p_{adj} < 0.05$, ** $p_{adj} < 0.01$ and **** $p_{adj} < 0.0001$.
